# Antibacterial and Antibiofilm Activity of *Aspergillus*-Derived Gliotoxin Against *Streptococcus pneumoniae* and *Staphylococcus aureus*: Interaction with Conventional Antibiotics

**DOI:** 10.3390/jof12090663

**Published:** 2026-09-02

**Authors:** Mirella Llamosí, Julio Sempere, Alicia Gómez-López, Jose Yuste, Mirian Domenech

**Affiliations:** 1 Spanish Pneumococcal Reference Laboratory, National Center for Microbiology, Instituto de Salud Carlos III, 28220 Madrid, Spain; mirella.llamosi@externos.isciii.es (M.L.); jsempere@isciii.es (J.S.); jyuste@isciii.es (J.Y.); 2 CIBER de Enfermedades Respiratorias, Instituto de Salud Carlos III, 28029 Madrid, Spain; 3 Mycology Reference and Research Laboratory, National Center for Microbiology, Instituto de Salud Carlos III, 28220 Madrid, Spain; aliciagl@isciii.es; 4 CIBER de Enfermedades Infecciosas, Instituto de Salud Carlos III, 28029 Madrid, Spain

**Keywords:** gliotoxin, *Aspergillus fumigatus*, *Streptococcus pneumoniae*, *Staphylococcus aureus*

## Abstract

Antimicrobial resistance and the formation of biofilms in Gram-positive pathogens such as *Streptococcus pneumoniae* and *Staphylococcus aureus* are making the treatment of infections increasingly difficult. This study systematically evaluates the effect of gliotoxin (GT), an antimicrobial metabolite produced by *Aspergillus fumigatus*, on bacterial growth and biofilm formation, as well as its activity in combination with antibiotics. Three strains of *S. pneumoniae* and two strains of *S. aureus* (one methicillin-susceptible and one methicillin-resistant) were analysed. Planktonic growth was examined, and biofilm prevention assays were conducted by assessing biomass via crystal violet staining and viable cell counts. In addition, the combined effect of GT with cefotaxime and vancomycin was evaluated. GT inhibited planktonic growth and significantly reduced the biomass and viability of biofilms in a concentration-dependent manner. Moreover, its combination with conventional antibiotics (cefotaxime or vancomycin) showed strong synergy, drastically decreasing bacterial survival. These findings highlight the potent antibacterial and anti-biofilm activity of GT and its ability to enhance antibiotic efficacy, providing insight into fungal–bacterial interactions and suggesting potential therapeutic applications.

## 1. Introduction

Antimicrobial resistance represents one of the most urgent threats to global health in the 21st century, because it reduces the effectiveness of standard treatments and promotes persistent or recurrent infections that were previously controllable with conventional antibiotics [1].

In this context, *Streptococcus pneumoniae* and *Staphylococcus aureus* stand out as two clinically critical Gram-positive pathogens. *S. pneumoniae* remains one of the leading causes of community-acquired pneumonia, meningitis, bacteremia, and otitis media worldwide, particularly affecting young children, older adults, and immunocompromised individuals [2,3]. Despite the widespread implementation of pneumococcal conjugate vaccines, pneumococcal disease continues to represent a major public health burden due to the emergence of non-vaccine serotypes and the increasing prevalence of antimicrobial-resistant lineages [4,5]. Resistance to β-lactams, macrolides, and other antimicrobial classes has been reported globally, complicating treatment in both hospital and community settings [6,7,8,9].

Similarly, *Staphylococcus aureus* is a major cause of skin and soft tissue infections, bacteremia, endocarditis, pneumonia, and device-associated infections worldwide. Recent studies continue to identify *S. aureus* as one of the leading bacterial causes of infectious disease-related morbidity and mortality in adults [10]. In particular, methicillin-resistant *S. aureus* (MRSA) remains a major public health concern due to its capacity to acquire multidrug resistance and cause both healthcare-associated and community-acquired infections [11]. Recent global burden analyses have shown that MRSA is among the antimicrobial-resistant pathogens responsible for the greatest number of deaths worldwide, with MRSA-associated mortality increasing substantially over the last three decades. Furthermore, S. aureus bacteremia remains associated with high mortality rates, causing an estimated 300,000 deaths annually worldwide [12]. The remarkable adaptive capacity of *S. aureus*, together with its ability to form biofilms, evade host immune responses, and acquire resistance determinants, has made it a priority pathogen for the development of new antimicrobial strategies [13].

One of the pathophysiological mechanisms underlying pathogenicity and antimicrobial resistance in these species is the formation of biofilms—multicellular communities immersed in an extracellular matrix that acts as a physical and physiological barrier against antimicrobial agents and immune responses [14,15,16,17]. This configuration not only promotes antibiotic tolerance by limiting their penetration but also produces metabolic changes that reduce the activity of drugs targeting active cellular processes [18]. Biofilms develop through organized stages of adhesion, growth, and maturation that are finely regulated by signaling networks such as quorum sensing, which modulate the expression of virulence and resistance factors [19]. In the case of *S. aureus*, this ability is a hallmark of chronic infections associated with implants and skin and soft tissues [20]. In *S. pneumoniae*, biofilm also plays a role in chronic colonization of the nasopharynx and the lower respiratory tract in patients with chronic obstructive pulmonary disease (COPD) and may facilitate coexistence with other pathogens in mixed niches, contributing to complex phenomena of resistance and persistence [21,22,23].

In natural and clinical environments, especially in the respiratory tract, these bacterial biofilms do not exist in isolation but interact with other microorganisms, including opportunistic fungi such as *Aspergillus fumigatus*. This filamentous fungus can establish fungal colonization, producing a wide array of secondary metabolites, including gliotoxin (GT), a compound belonging to the epidiotioxopiperazine family with multifunctional biological properties [24].

It has been shown that GT and other metabolites of *A. fumigatus* have effects on microorganisms and host cells, including the induction of apoptosis, the suppression of immune responses, and the alteration of metabolic processes essential for cell survival [25]. However, their specific interaction with Gram-positive bacterial pathogens or their effect on biofilm processes is not yet fully defined [26].

In this context, this study evaluates the effects of GT produced by *A. fumigatus* on the planktonic growth and biofilm formation of *S. pneumoniae* and *S. aureus*. Specifically, we aim to determine whether GT modulates bacterial viability, biofilm biomass and/or the structural architecture of these communities, as well as to explore its possible impact on biofilm-associated antimicrobial tolerance.

## 2. Materials and Methods

### 2.1. Strains, Media and Culture Conditions

*S. pneumoniae* and *S. aureus* strains were used for this study as clinically relevant Gram-positive bacteria (Table 1). Three strains of *S. pneumoniae* were included: one unencapsulated strain (R6) and two isogenic mutants (YNM4 and YNM12) corresponding to serotypes 3 and 19A, respectively. For *S. aureus*, two clinical isolates were analysed, including one methicillin-susceptible strain (MSSA) (60031/19) and one methicillin-resistant strain (60061/19).

For liquid medium culture, the pneumococcal strains were grown in CpH8 medium supplemented with 0.08% yeast extract (C + Y medium); *S. aureus* strains were grown in Tryptic Soy Broth medium supplemented with glucose (0.4%) and yeast extract (0.3%) (TSBGY medium) [27]. Growth was controlled by measuring the optical density at 550 nm (*A*_550_). All strains were preserved in their specific medium and stored at −80 °C.

### 2.2. Gliotoxin

Commercial GT from Enzo Life Sciences (BML-PI129-0010-10 mg; CAS number 67-99-2, Farmingdale, NY, USA) with a purity of 97% was used for the assays. This GT was solubilised in dimethyl sulfoxide (DMSO) and a stock solution was prepared and frozen at −20 °C.

### 2.3. Antibiotics

Two antibiotics widely used in clinical practice were used. Cefotaxime sodium salt (C7039-1G, Sigma, Saint Louis, MO, USA), a third-generation cephalosporin used for pneumococcal infections and MSSA. Vancomycin (V2002-250MG, Sigma), a glycopeptide widely used for MRSA infections. Both antibiotics were prepared in distilled water and the stock solutions were stored at −20 °C.

### 2.4. Determination of Minimum Inhibitory Concentration (MIC)

The Minimum Inhibitory Concentrations (MICs) of cefotaxime (CTX) for *S. pneumoniae* and vancomycin (VAN) for *S. aureus* were determined by broth microdilution according to the recommendations of the European Committee on Antimicrobial Susceptibility Testing (EUCAST) (Table 2). Serial two-fold dilutions of each antibiotic were prepared in the corresponding culture medium and inoculated with standardized bacterial suspensions. After incubation under the appropriate conditions for each species, the MIC was defined as the lowest antibiotic concentration that completely inhibited visible bacterial growth.

### 2.5. Growth Curves

Pneumococcal cells were grown to an optical density of 0.5–0.6 at *A*_550_, diluted 1:100, and 200 µL were added per well to a 96-well polystyrene microtiter plate (Falcon 3095; Corning; Corning, NY, USA). Different concentrations of GT, dissolved in DMSO, were added at the beginning of the assay. Two control conditions were included: one containing DMSO without GT and another containing neither GT nor DMSO. Plates were then incubated at 37 °C in an Epoch 2 microplate reader (BioTek, Winooski, VT, USA), which functioned as an incubator and measured absorbance at *A*_550_ every 30 min.

### 2.6. Biofilm Inhibition

Biofilm formation was performed using 96-well polystyrene microtiter plates. The strains were incubated at 37 °C in the corresponding medium (TSBGY or C + Y) to an optical density of 0.5–0.6 at *A*_550_. The culture was diluted to the optical density previously determined to correspond to approximately 4.5 × 10^6^ CFU/mL, based on a calibration curve relating OD measurements to viable cell counts obtained by plate counting. Then, 200 μL per well was added to a 96-well polystyrene microtiter plate (Costar 3595; Corning; Corning, NY, USA) and different concentrations of GT were added (0.05 µg/mL; 0.1 µg/mL; 0.5 µg/mL; 1 µg/mL; 2 µg/mL; 4 µg/mL; 6 µg/mL; 8 µg/mL). Plates were incubated for 5 h at 34 °C for pneumococcal strains and 24 h at 37 °C for *S. aureus* strains. After incubation, in the case of *S. pneumoniae*, the biofilm was processed by staining with crystal violet. Briefly, total growth was measured using the BioTek Epoch 2 reader and then crystal violet (1%) was added for 15 min, washed three times with distilled water, solubilised with ethanol and then the biofilm biomass was measured at 595 nm (*A*_595_). For the *S. aureus* biofilm, the supernatant was discarded, 200 µL of water and 50 µL of CV (1%) were added and the biofilm was incubated for 15 min. After two washes with distilled water, the biofilm was solubilised with ethanol, and absorbance was measured at *A*_595_. In addition, biofilms were analysed by viable cell count. Biofilm was resuspended in 100 µL of distilled water, serial dilutions were made in PBS (1×), and 10 µL of each dilution was plated onto Mueller–Hinton agar with 5% blood.

In the assays combining the GT and antibiotics (cefotaxime or vancomycin), the same procedure was followed as for the biofilm inhibition assays, but with the addition of these antibiotics together with the GT. CTX was used for the *S. pneumoniae* biofilm and VAN for the *S. aureus* biofilm. In both cases, a concentration corresponding to the MIC (0.016 µg/mL for CTX in the case of *S. pneumoniae* strains and 4 µg/mL for VAN in the case of *S. aureus* strains) was used.

### 2.7. Statistical Analysis

Data represent results obtained from at least three repeated independent experiments, with at least three replicates. Statistical analyses were performed using GraphPad InStat v. 8.0 (GraphPad Software). For comparison of two groups, we used the two-tailed Student’s *t* test. For multiple comparisons, we chose a one-way ANOVA test and a Dunnett’s post hoc test. Differences were considered statistically significant at *, *p* < 0.05; **, *p* < 0.01; ***, *p* < 0.001.

## 3. Results

### 3.1. Effect of Gliotoxin on Planktonic Growth of S. pneumoniae and S. aureus

**Figure 1:**
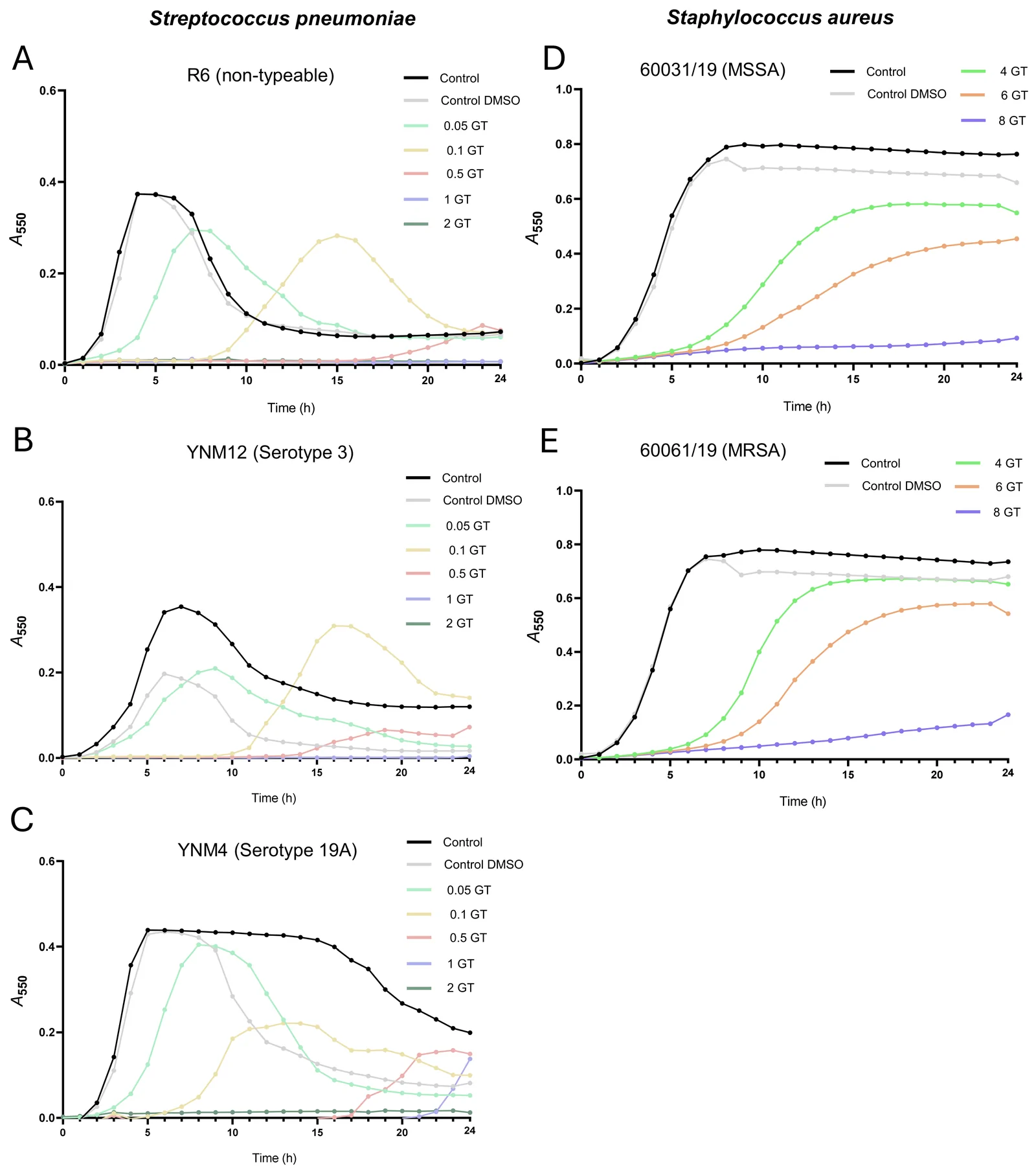
Inhibition of planktonic growth of *S. pneumoniae* and *S. aureus* with different concentrations of GT. Representative growth curves of *S. pneumoniae* (left panels, pink) and *S. aureus* (right panels, green) in the presence of different concentrations of GT. Each graph shows bacterial growth over 24 h at 37 °C compared with the untreated control (black line) and the DMSO control (grey line). (**A**) Growth inhibition of unencapsulated *S. pneumoniae* (R6 strain). (**B**) Growth inhibition of serotype 3 *S. pneumoniae* (YMN12 strain). (**C**) Growth inhibition of serotype 19A *S. pneumoniae* (YNM4 strain) by GT. (**D**) Growth inhibition of MSSA (60031/19 strain). (**E**) Growth inhibition of MRSA (60061/19 strain).

Growth-inhibition curves were plotted to investigate the effect of GT on the growth of both bacteria. In the case of the unencapsulated strain of *S. pneumoniae*, complete growth inhibition was observed from a concentration of 0.5 µg/mL. At lower concentrations, a delay in the exponential growth phase was observed, with growth starting at 3 h when the GT concentration was 0.05 µg/mL and at 9 h when the concentration was 0.1 µg/mL (Figure 1A). When the strain used displays a serotype 3 capsule, lower growth is observed at low concentrations of GT compared with the control, and complete growth inhibition is observed at the highest concentration of GT (Figure 1B). If the capsule is serotype 19A, a shortening of the stationary phase is observed at the lowest concentration of GT, whereas at higher concentrations, a delay in growth is again observed, with complete inhibition at a concentration of 2 µg/mL (Figure 1C). When these curves are analyzed against *S. aureus* strains, a delay in growth is observed from the lowest concentration used (4 µg/mL), reaching almost complete inhibition at 8 µg/mL of GT in MSSA and MRSA strains (Figure 1D,E).

Furthermore, growth-inhibition curve assays were also carried out using the non-toxic GT derivative, bis(methylthio)gliotoxin, another secondary metabolite produced during infection by *A. fumigatus* [28]. No growth-inhibitory activity was detected against any of the bacterial species studied.

### 3.2. Gliotoxin Impairs Biofilm Biomass Formation

**Figure 2:**
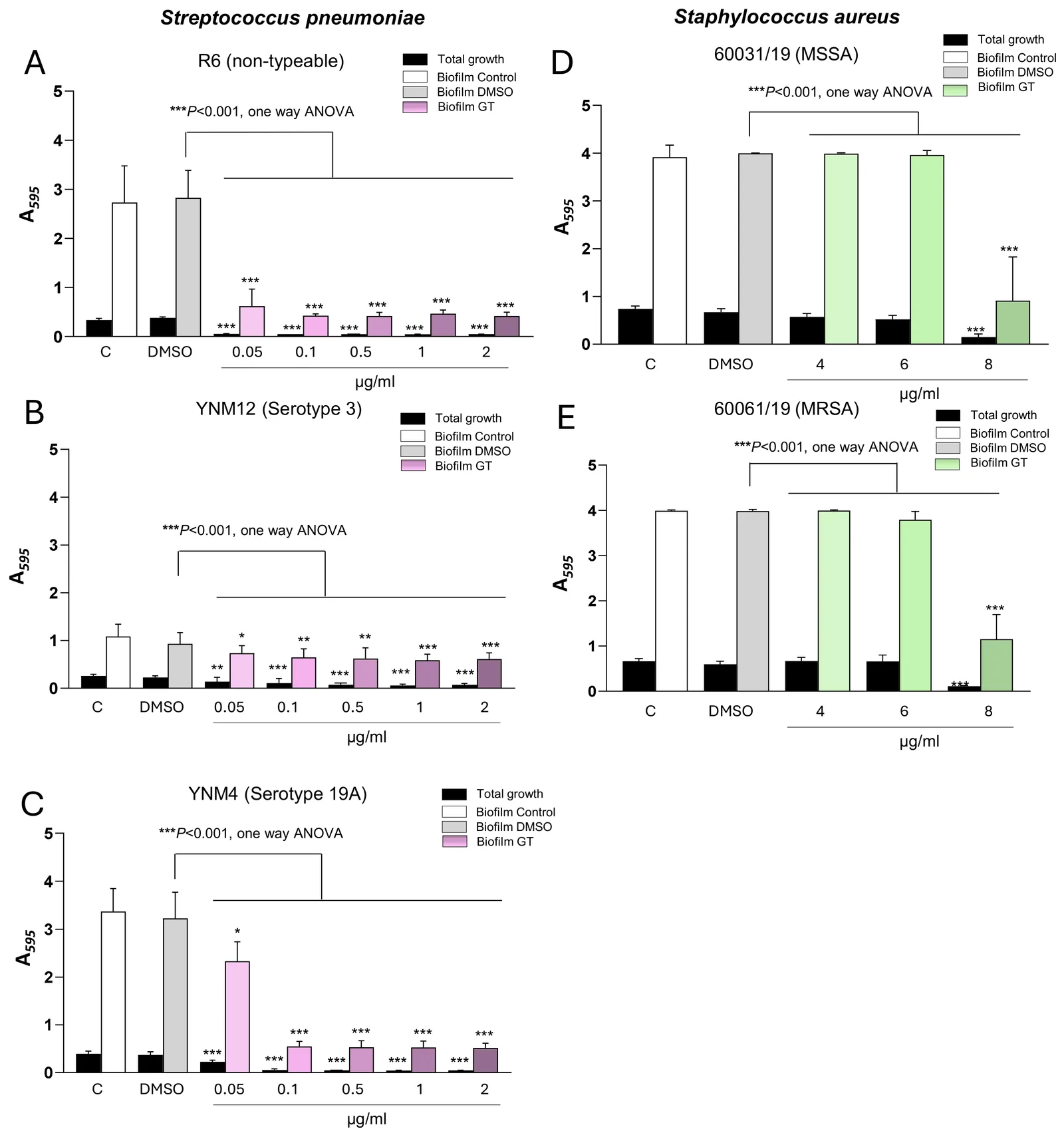
Inhibition of *S. pneumoniae* and *S. aureus* biofilms by increasing concentrations of GT. Different concentrations of GT were added at the time of inoculation, and the biofilms were incubated for 5 h at 37 °C for *S. pneumoniae* and for 24 h at 37 °C for *S. aureus*. Then, the biofilms were quantified using CV staining. For *S. pneumoniae*, the unencapsulated strain R6 (**A**), the serotype 3 strain YNM12 (**B**), and the serotype 19A strain YNM4 (**C**) were analyzed. For *S. aureus*, an MSSA strain (60031/19) (**D**) and an MRSA strain (60061/19) (**E**) were used. Black bars represent total bacterial growth (adherent (biofilm) plus non-adherent (planktonic) cells), whereas white/colored bars represent biofilm biomass. Data represent the mean of three independent experiments. Error bars indicate standard deviation. Asterisks denote statistically significant differences compared with the DMSO control (two-tailed Student’s *t*-test: *, *p* < 0.05; **, *p* < 0.01; ***, *p* < 0.001). For multiple comparisons of biofilm biomass, we performed one-way ANOVA followed by a Dunnett’s post hoc test.

The effect of GT on the biofilm biomass was assessed in the different strains of *S. pneumoniae* and *S. aureus*. Treatment with GT inhibited both the growth and biofilm formation of *S. pneumoniae* (Figure 2A–C). In all three strains, concentrations as low as 0.05 µg/mL significantly reduced bacterial growth and biofilm biomass, with the most pronounced effect observed in the R6 strain (Figure 2A). In the unencapsulated (R6) strain and in the serotype 3 strain (YNM12), maximal inhibition was achieved at 0.05 µg/mL GT, with no significant additional effect at higher concentrations. In contrast, the serotype 19A strain (YNM4) showed maximal inhibition of both growth and biofilm formation at 0.1 µg/mL GT (Figure 2C). These results suggest that the capsular type may influence the preventive effect of GT on pneumococcal biofilm formation only at lower concentrations of GT, whereas at concentrations ≥ 0.1 µg/mL the effect was similar irrespective of the capsule.

In *S. aureus*, we observed a lower antimicrobial effect compared with *S. pneumoniae* (Figure 2D,E). In both biofilms by the MSSA and MRSA strains, bacterial growth remained virtually unchanged at concentrations of up to 6 µg/mL. Only at the highest concentration tested (8 µg/mL) did we observe a significant decrease in growth and biofilm formation in both strains. In both cases, one-way ANOVA for multiple comparisons revealed significant differences among groups (*p* < 0.001), and post hoc Dunnett’s test showed that only the group treated with 8 µg/mL differed significantly from the control group (Figure 2D,E).

Furthermore, biofilm inhibition assays were also carried out using the non-toxic GT derivative, bis(methylthio)gliotoxin, but no effect was observed compared with the control in any of the bacteria studied. Both GT and the non-toxic GT derivative were used in biofilm disaggregation assays (treatment after biofilm development) and did not have any effect on the mature biofilm.

### 3.3. Effect of Gliotoxin on the Viability of Biofilm-Associated Cells

**Figure 3:**
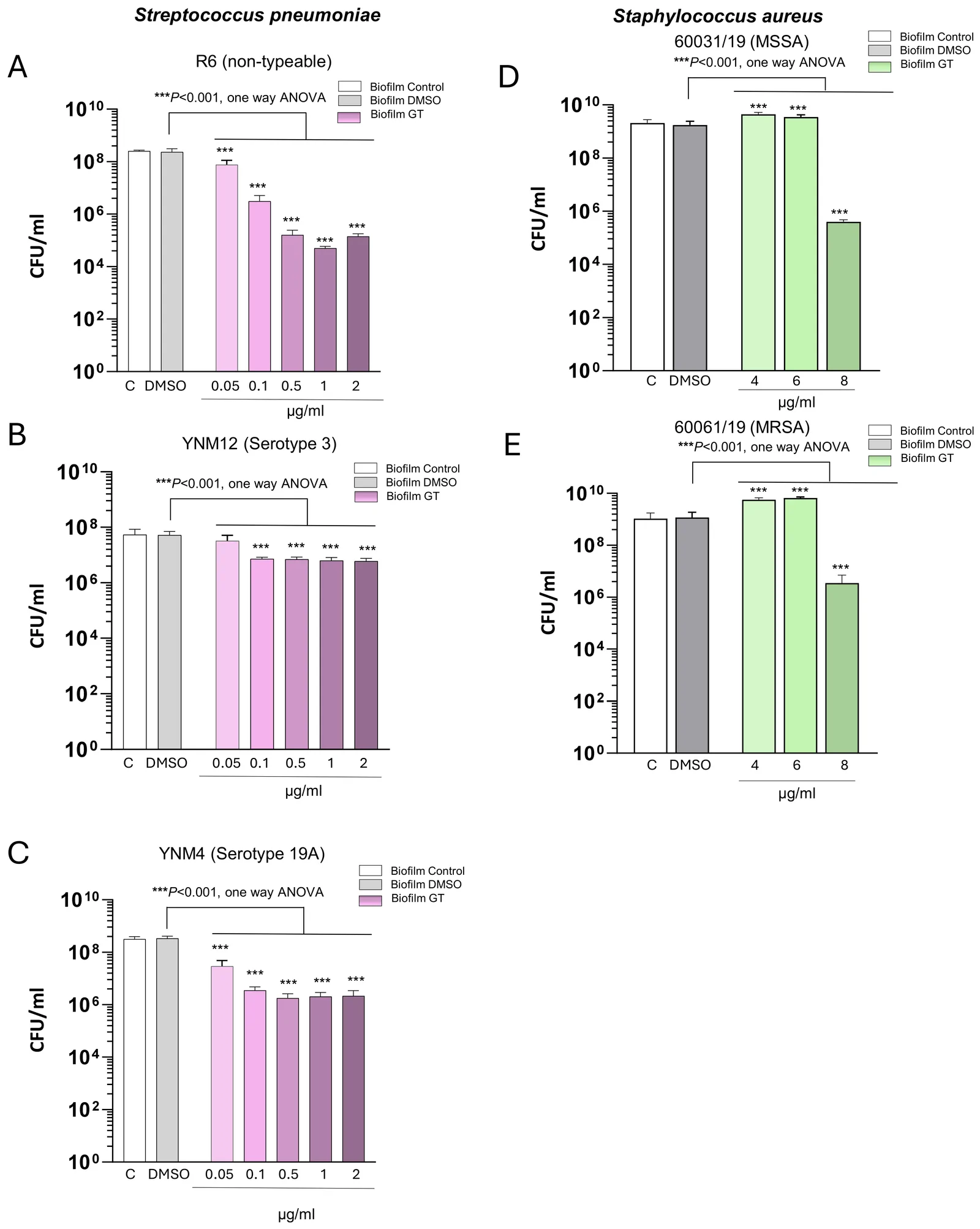
Effect of GT on *S. pneumoniae* and *S. aureus* biofilm viability. Different concentrations of GT were added at the time of inoculation, and the biofilms were incubated for 5 h at 37 °C for *S. pneumoniae* and for 24 h at 37 °C for *S. aureus*. Then, biofilms were processed for viable cell counts. For *S. pneumoniae*, the unencapsulated strain R6 (**A**), the serotype 3 strain YNM12 (**B**), and the serotype 19A strain YNM4 (**C**) were analyzed. For *S. aureus*, an MSSA strain (60031/19) (**D**) and an MRSA strain (60061/19) (**E**) were used. Data represent the mean of three independent experiments. Error bars indicate standard deviation. Asterisks denote statistically significant differences compared with the DMSO control (two-tailed Student’s *t*-test: ***, *p* < 0.001). For multiple comparisons, we performed one-way ANOVA followed by a Dunnett’s post hoc test.

To evaluate the impact of GT on bacterial viability, CFU/mL were quantified following exposure to different concentrations of the compound (Figure 3). In *S. pneumoniae* (Figure 3A–C), GT caused a clear, concentration-dependent reduction in the number of viable cells within biofilms for all strains tested. In the unencapsulated strain, a decrease in CFU was observed at concentrations as low as 0.05 µg/mL, with further reductions at higher concentrations. The serotype 3 strain YNM12 exhibited a more moderate response, although significant reductions in viable cells were still evident compared with the control. In contrast, the serotype 19A strain YNM4 displayed an intermediate sensitivity, with a pronounced decrease in biofilm-associated viable cells from 0.05–0.1 µg/mL onward. The strongest preventive effect of GT on pneumococcal biofilms was detected in the R6 strain at 1 µg/mL, where biofilm-associated viable cells were reduced by approximately three orders of magnitude compared with the control without GT. These results suggest that the presence and type of capsule may influence the preventive effect of GT on pneumococcal biofilm formation.

In *S. aureus* (Figure 3D,E), both the MSSA and the MRSA strains showed a lower susceptibility to GT compared with *S. pneumoniae*. Viable cell counts remained largely unchanged at concentrations up to 6 µg/mL, and a significant reduction in CFU was only detected at the highest concentration tested (8 µg/mL). Additionally, one-way ANOVA for multiple comparisons revealed significant differences among groups (*p* < 0.001). Overall, these results indicate that GT effectively reduces the number of viable cells within pneumococcal biofilms at low concentrations, whereas *S. aureus* biofilms require substantially higher concentrations for a comparable effect.

### 3.4. Combined Treatment with Gliotoxin and Antibiotic Enhances Biofilm Inhibition

**Figure 4:**
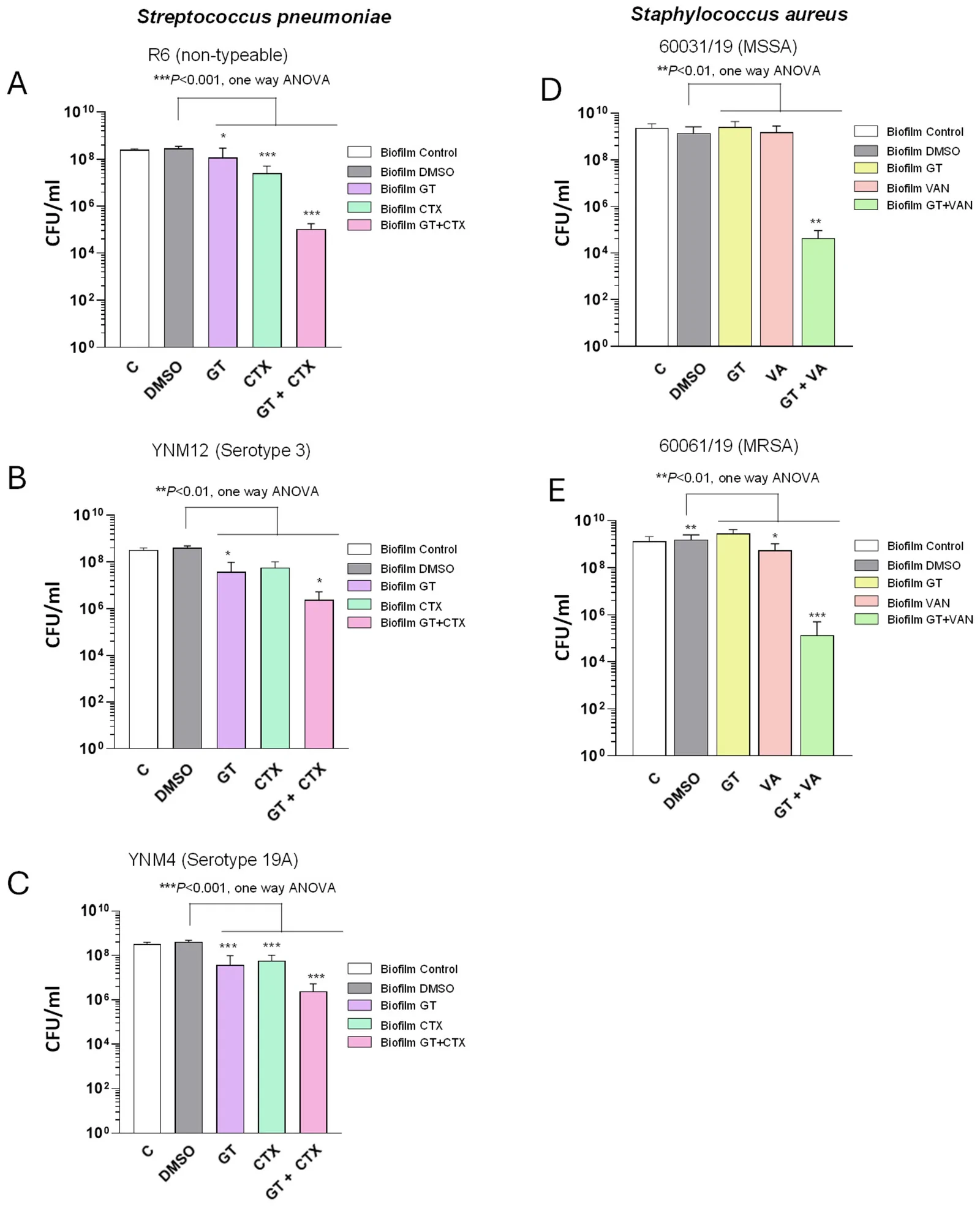
Inhibition of *S. pneumoniae* and *S. aureus* biofilms with a combination of GT and an antibiotic. The corresponding concentrations of GT (0.1 µg/mL or 4 µg/mL) and the antibiotic (sub-MIC: CTX (0.004 µg/mL) or VAN (2 µg/mL)) were added at the time of inoculation, and biofilms were incubated at 37 °C for 5 h for *S. pneumoniae* and for 24 h for *S. aureus*. Biofilm viability was subsequently assessed by viable cell counting. For *S. pneumoniae*, the unencapsulated strain R6 (**A**), the serotype 3 strain YNM12 (**B**), and the serotype 19A strain YNM4 (**C**) were used. For *S. aureus*, an MSSA strain (60031/19) (**D**) and an MRSA strain (60061/19) (**E**) were analyzed. Data represent the mean of three independent experiments. Error bars indicate standard deviation. Asterisks denote statistically significant differences compared with the DMSO control (two-tailed Student’s *t*-test: *, *p* < 0.05; **, *p* < 0.01; ***, *p* < 0.001). For multiple comparisons, we performed one-way ANOVA followed by a Dunnett’s post hoc test.

In order to assess the potential combined effects of the compound with antibiotics, bacterial viability was determined following individual and combined treatments against biofilms, as these bacterial stages are more difficult to treat with antibiotics, and the results above showed lower antimicrobial effects by GT compared with planktonic cultures (Figure 4).

In *S. pneumoniae*, the combination of the compound with CTX enhanced the antibacterial effect in all strains analysed (Figure 4A–C). In strain R6, treatment with GT or CTX alone produced a moderate reduction in viability of biofilm; however, the combination of both resulted in a significantly greater decrease in CFU/mL, reaching levels several orders of magnitude lower than the individual treatments. A similar pattern was observed in strain YNM12 (serotype 3), where the GT + CTX combination induced a more pronounced reduction compared with each treatment alone. In strain YNM4 (serotype 19A), although CTX alone showed a limited effect, the combination with GT resulted in a significant decrease in bacterial viability, demonstrating a potential enhancing effect.

In *S. aureus*, the combined effect of GT with VAN was also evaluated (Figure 4D,E). In the MSSA strain, individual treatments with GT or VAN did not produce a substantial reduction in bacterial viability at the concentrations tested; however, their combination resulted in a significant decrease in viable cells. Similarly, in the MRSA strain, the GT + VAN combination caused a marked reduction in bacterial viability, exceeding the effect observed with either compound alone, whereas VAN by itself showed only a moderate effect. Biofilm viability was reduced by approximately three orders of magnitude in MRSA and four orders of magnitude in MSSA following treatment with the GT + VAN combination.

Overall, these results indicate that the GT compound enhances the antimicrobial effect of conventional antibiotics, such as CTX or VAN, used at sub-inhibitory concentrations, suggesting a potential enhancing interaction that significantly increases bactericidal activity against both bacterial species.

## 4. Discussion

This study provides evidence that GT, a secondary metabolite produced by *A. fumigatus*, can influence bacterial growth and biofilm formation in relevant clinically Gram-positive bacteria such as *S. pneumoniae* and *S. aureus*, with distinct responses observed between species.

Consistent with this observation, GT reduced bacterial growth in both species, with pneumococci showing susceptibility at lower concentrations (0.05–2 µg/mL), whereas *S. aureus* required higher doses (4–8 µg/mL). This differential sensitivity aligns with previous reports describing the antibacterial activity of GT against both Gram-positive and Gram-negative bacteria [29]. At the molecular level, recent work by Downes et al. (2023) demonstrated that GT induces intracellular zinc depletion, leading to widespread disruption of bacterial enzymatic and regulatory processes, highlighting metal homeostasis as a key antimicrobial target [30].

In addition to metal chelation, GT is a redox-active epipolythiodioxopiperazine capable of generating reactive oxygen species and forming disulfide bonds with thiol-containing proteins, leading to enzyme inactivation and disruption of cellular redox balance [31]. In bacteria, oxidative stress damages essential cellular components, particularly proteins and enzymes, leading to impaired metabolic processes and growth inhibition [32]. These combined mechanisms—metal ion depletion, oxidative stress induction, and thiol modification—may contribute synergistically to the antibacterial effects observed in this study. The higher sensitivity of pneumococcus at lower concentrations may reflect its more limited antioxidant defenses, whereas *S. aureus* appears to tolerate better low levels of stress but becomes susceptible at higher GT concentrations. Notably, to date, there is a lack of studies directly evaluating the effect of GT on *S. pneumoniae*, which highlights the novelty of our findings. Most available evidence for the antibacterial activity of GT has been obtained in other bacterial species, including *S. aureus* or *Enterococcus faecalis* and Gram-negative pathogens such as *Salmonella enterica* [30,33,34]. These studies have also shown that GT can inhibit bacterial growth through multiple mechanisms, including intracellular metal ion depletion and disruption of thiol-dependent processes [35]. Although these mechanisms have not been explicitly demonstrated in pneumococcus, they may be conserved across different bacterial species. Therefore, the growth inhibition observed in pneumococcus in our study could be attributable to similar processes, including redox imbalance, thiol modification, and disruption of metal-dependent enzymatic pathways; however, this hypothesis remains to be experimentally validated.

Beyond its antibacterial activity, GT was found to significantly modulate biofilm formation in both species. This observation aligns with growing evidence that microbial secondary metabolites can function as interkingdom signaling molecules, influencing bacterial behavior, including biofilm formation and community organization [36].

In *S. pneumoniae*, biofilm formation is closely associated with the production of extracellular DNA, a major structural component of the biofilm matrix that is partly derived from bacterial lysis [15]. In *S. aureus*, biofilm development is regulated by complex networks including *agr* and *sarA*, which control virulence and biofilm-associated phenotypes [37]. Therefore, GT could potentially influence biofilm dynamics through interference with redox-sensitive regulatory pathways. However, the molecular mechanisms responsible for the antibiofilm effects observed in the present study were not investigated and remain to be determined. Notably, the effects observed in the MRSA strain are of great relevance. MRSA strains are characterized by reduced susceptibility to β-lactam antibiotics and a strong association with biofilm-mediated persistence, which complicates treatment [16,38]. The observed impact of GT on both bacterial growth and biofilm formation in this strain suggests that its activity extends to antibiotic-resistant bacteria.

An additional relevant finding of this study is the differential effect of GT across distinct *S. pneumoniae* serotypes. The inclusion of strains with different capsular polysaccharides but containing an identical genetic background allowed us to assess the contribution of the polysaccharide capsule to GT susceptibility. This is an important aspect because in recent years it has been demonstrated that the pneumococcal background in terms of sequencetypes/lineages have different phenotypes of host immune response, antimicrobial resistance, vaccine susceptibility and biofilm formation [39,40,41]. Notably, non-encapsulated strains exhibited increased sensitivity to GT compared with their encapsulated counterparts, suggesting that the capsule may confer a protective effect against GT or interfere with the pneumococcal target site of GT. The pneumococcal capsule is a major virulence factor that plays a central role in immune evasion, particularly by inhibiting phagocytosis and complement-mediated clearance [42,43]. In this context, its presence may also limit the interaction of GT with the bacterial surface or reduce its intracellular effects. These observations are consistent with the role of the capsule as a physical and functional barrier and further support its importance not only in host–pathogen interactions but also in modulating susceptibility to antimicrobial compounds [44]. Although the precise mechanisms underlying this differential susceptibility remain to be elucidated, they may involve reduced GT penetration or altered redox dynamics in encapsulated strains. GT has been described as a neutral, hydrophobic molecule [45], and differences have been observed in the charges of the various capsular polysaccharides, with serotype 3 being the most electronegative [46,47,48,49,50]. This could explain the differences observed in the effect of GT.

At a broader level, the capacity of GT to modulate bacterial physiology and biofilm architecture may enhance the susceptibility of resistant bacteria to antimicrobial agents. Although the underlying mechanisms are unknown, they likely involve redox imbalance and disruption of essential cellular processes that are not directly targeted by conventional antibiotics. These findings highlight the potential relevance of gliotoxin or gliotoxin-inspired approaches in the context of infections caused by multidrug-resistant pathogens. The interaction between GT and antibiotics observed in this study further supports this modulatory role. Previous studies have shown that GT exhibits antibacterial activity against *S. aureus* and may enhance antimicrobial efficacy under certain conditions [30,31,34,51]. In addition, biofilm modulation may alter the structural properties of the matrix, which can influence antibiotic penetration and thereby affect treatment efficacy [52]. However, whether any of these mechanisms are responsible for the enhanced activity observed in the present study was not investigated and remains to be determined.

From a clinical perspective, these findings are particularly relevant in polymicrobial infections of the respiratory tract, where *A. fumigatus* frequently coexists with bacterial pathogens. Fungal-derived metabolites such as GT have been increasingly recognized as key mediators of interkingdom interactions capable of shaping microbial communities leading to disease progression [53]. The ability of GT to modulate both bacterial growth and biofilm formation suggests that it may contribute to bacterial persistence, impairing treatment outcomes in coinfections.

Despite these promising observations, the therapeutic application of GT is limited by its well-documented toxicity. GT is a potent immunosuppressive molecule capable of inducing apoptosis in immune cells and disrupting host defenses [35,54,55]. The lowest tested concentration (0.05 µg/mL, ~150 nM) is slightly above the range where cytotoxic effects have been reported (>100 nM), indicating a narrow therapeutic window between antibiofilm efficacy and host toxicity [31]. Therefore, while GT itself is unlikely to be used as a therapeutic agent, our study showing its antimicrobial activity against relevant multidrug-resistant bacteria such as pneumococcus and MRSA, including biofilms, will be useful for exploring alternative derivatives with lower toxicity. Other limitations include the fact that the antibiofilm activity of gliotoxin was evaluated using crystal violet staining and viable cell counts, which provide information on biofilm biomass and culturable biofilm-associated cells but do not allow assessment of biofilm architecture, metabolic activity, or phenotypic heterogeneity within the bacterial population. In addition, the *S. aureus* model focused exclusively on surface-attached biofilms and did not evaluate pellicle-associated cells at the air–liquid interface. Moreover, GT and the non-toxic derivative did not have any effect in the mature biofilm, so GT could only be used as a preventive agent. Finally, the number of strains included in this study was limited and was selected to represent clinically relevant phenotypes, including different pneumococcal capsular backgrounds and both methicillin-susceptible and methicillin-resistant *S. aureus* isolates. Therefore, caution should be exercised when extrapolating these findings to the broader diversity of pneumococcal and staphylococcal populations. The inclusion of a larger collection of clinical isolates and reference strains will be important to further validate and generalize the findings reported here.

Future studies should focus on elucidating the molecular basis of gliotoxin activity through transcriptomic, proteomic, and metabolomic approaches. Investigating the role of oxidative stress, metal homeostasis, and quorum sensing pathways will be essential. Also, these future studies should employ complementary methodologies and more complex biofilm models to further characterize the effects of gliotoxin on biofilm development. Approaches such as metabolic activity assays, advanced imaging techniques, and air–liquid interface or colony biofilm models will be valuable to evaluate biofilm architecture, metabolic activity, phenotypic heterogeneity, persister-like populations, and pellicle-associated cells. In addition, further work will be required to elucidate the molecular mechanisms underlying the phenotypes observed in the present study. Additionally, validation in polymicrobial and in vivo models will be necessary to determine the clinical relevance of these findings.

In conclusion, this study demonstrates that gliotoxin inhibits bacterial growth, reduces biofilm biomass formation, and decreases the number of viable biofilm-associated cells in selected strains of *S. pneumoniae* and *S. aureus*, including MRSA. In addition, gliotoxin enhanced the activity of conventional antibiotics under the experimental conditions tested. These findings provide further evidence that fungal secondary metabolites can influence bacterial physiology and biofilm development, although the molecular mechanisms underlying these effects remain to be elucidated.

## Figures and Tables

**Table 1:** Strains used in this study with their description.

Bacteria	Strain	Description *
*Streptococcus pneumoniae*	R6	Unencapsulated [15]
*Streptococcus pneumoniae*	YNM12	Transformant M11 with DNA from strain 830/08; serotype 3 (This study)
*Streptococcus pneumoniae*	YNM4	Transformant M11 with DNA from strain 1228/19; serotype 19A [27]
*Staphylococcus aureus*	60031/19	MSSA; serotype 5 [27]
*Staphylococcus aureus*	60061/19	MRSA; serotype 5 [27]

* Source: serotype/reference.

**Table 2:** Minimum Inhibitory Concentration (MIC) values of the antibiotics used in the biofilm combination assays.

Bacterial Strain	Bacteria	Antibiotic	MIC (µg/mL)
R6	*S. pneumoniae*	CTX	0.016
YNM12	*S. pneumoniae*	CTX	0.016
YNM4	*S. pneumoniae*	CTX	0.016
60031/19	*S. aureus*	VAN	4
60061/19	*S. aureus*	VAN	4

## Data Availability

The raw data supporting the conclusions of this article will be made available by the authors upon request.

## Abbreviations

The following abbreviations are used in this manuscript:

CV	Crystal violet
C + Y	CpH8 medium supplemented with 0.08% yeast extract
CFU	Colony-Forming Unit
CTX	Cefotaxime
DMSO	Dimethyl sulfoxide
GT	Gliotoxin
MIC	Minimum Inhibitory Concentration
MRSA	Methicillin-Resistant *Staphylococcus aureus*
MSSA	Methicillin-Susceptible *Staphylococcus aureus*
PBS1x	Phosphate-Buffered Saline
TSBGY	Tryptic Soy Broth medium supplemented with glucose (0.4%) and yeast extract (0.3%)
VAN	Vancomycin
